# Biomarker-driven immunotherapy and adoptive cell therapy in recurrent pancreatic ductal adenocarcinoma

**DOI:** 10.3389/fmed.2026.1900701

**Published:** 2026-08-13

**Authors:** Tao He, Guoan Li, Fuzhen Qi

**Affiliations:** 1 Department of Hepatobiliary and Pancreatic Surgery, The Affiliated Huai’an No. 1 People’s Hospital of Nanjing Medical University, Huai’an, Jiangsu, China; 2 The Affiliated Huai’an No. 1 People’s Hospital of Nanjing Medical University, Huai’an, Jiangsu, China

**Keywords:** adoptive cell therapy, biomarker, Car-T, circulating tumor DNA, immunotherapy, molecular residual disease, pancreatic ductal adenocarcinoma, recurrence

## Abstract

Recurrent pancreatic ductal adenocarcinoma (PDAC) includes circulating tumor DNA (ctDNA)-defined molecular residual disease (MRD), isolated or oligometastatic relapse, disseminated measurable recurrence, and heavily pretreated refractory disease. These states differ in tumor burden, treatment urgency, tissue accessibility, and immune competence and should not be treated as one population. Immune checkpoint blockade has minimal activity in unselected PDAC, and randomized trials have not shown consistent benefit from adding checkpoint inhibitors to chemotherapy. However, rare microsatellite instability-high/mismatch repair-deficient (MSI-H/dMMR) tumors and selected antigen- or human leukocyte antigen (HLA)-defined subgroups support precision immune intervention. This Mini Review evaluates biomarkers for immunotherapy and adoptive cell therapy, including ctDNA kinetics, tumor mutational burden, KRAS and neoantigen profiles, antigen presentation and target-antigen density, effector immune state, myeloid and fibroblast-mediated suppression, and spatial immune exclusion. It compares checkpoint blockade, personalized and KRAS-directed vaccines, chimeric antigen receptor T-cell (CAR-T), chimeric antigen receptor natural killer-cell (CAR-NK), and T-cell receptor (TCR)-engineered therapies by recurrence setting, evidence maturity, feasibility, and failure mechanisms. Checkpoint blockade is currently most defensible for MSI-H/dMMR disease; vaccines and cell therapies remain investigational and are best tested in biomarker-selected trials, especially in low-burden or molecular recurrence. We propose a five-step workflow to classify recurrence, identify actionable biomarkers, match evidence-appropriate interventions, assess feasibility, and monitor response and immune escape. This framework supports trial design and disciplined clinical translation rather than empiric immunotherapy in unselected recurrent PDAC.

## 1 Introduction

Pancreatic ductal adenocarcinoma (PDAC) is among the most lethal solid tumors, with high rates of occult dissemination, postoperative relapse, and resistance to systemic therapy (1, 2). Even after macroscopically complete resection and adjuvant therapy, recurrence remains common, and early relapse is associated with particularly poor survival (3, 4). Recurrent PDAC is not a single clinical state. It ranges from ctDNA-positive molecular residual disease without radiographic lesions to isolated or oligometastatic recurrence, disseminated measurable disease, and heavily pretreated refractory progression. These states differ in tumor burden, tempo, prior treatment exposure, biopsy feasibility, and the time available for an immune response to develop. These recurrence-pattern and MRD distinctions are supported by postoperative relapse and ctDNA studies (3–7).

Immunotherapy has transformed several solid tumors, but unselected PDAC has remained largely refractory. Single-agent CTLA-4 blockade produced no conventional objective responses in a phase 2 study, PD-L1 blockade with or without CTLA-4 inhibition yielded response rates of 0 and 3.1%, respectively, and the randomized PA.7 trial found no survival benefit from adding durvalumab and tremelimumab to gemcitabine plus nab-paclitaxel (8). The randomized PRINCE study generated a signal in one nivolumab-containing arm relative to a historical benchmark, but benefit was not consistent across arms, the study was not powered for between-arm comparisons, and the correlative biomarkers were exploratory (9). These results show that immune activation alone is usually insufficient in PDAC and that treatment selection must account for antigenicity, immune-cell access, stromal and myeloid suppression, disease burden, and treatment timing. These trial data define the evidence boundary for unselected PDAC (8–11).

Nevertheless, recurrent PDAC is immunologically heterogeneous. Rare tumors with microsatellite instability-high or mismatch repair deficiency (MSI-H/dMMR), selected KRAS or other actionable neoantigens, persistent ctDNA, preserved antigen presentation, sufficiently homogeneous surface-antigen expression, or less suppressive immune niches may be candidates for biomarker-directed immune strategies (5–7, 12–14). The relevant question is therefore not whether immunotherapy works uniformly in recurrent PDAC, but which recurrence state, biomarker, and therapeutic mechanism can be matched with sufficient biological and clinical justification.

Recent reviews have surveyed pancreatic cancer immunotherapy broadly, but many combine resected, treatment-naive metastatic, recurrent, and refractory populations and present emerging platforms without consistently distinguishing evidence maturity. This can obscure clinically important differences in tumor burden, prior therapy, immune fitness, tissue availability, and manufacturing time. The present Mini Review is focused specifically on recurrent PDAC and adds value by: (i) separating molecular, oligometastatic, disseminated, and refractory recurrence; (ii) comparing positive and negative clinical evidence; (iii) distinguishing clinically actionable, early-phase, and exploratory biomarkers; and (iv) translating these distinctions into a practical trial-oriented workflow. This focused framing is grounded in current biomarker, immunotherapy, and PDAC treatment literature (15–21). For this revision, PubMed/MEDLINE was searched through July 2026, focusing on studies from 2021 onward; reference lists of key trials and reviews were also screened. Search terms included PDAC, recurrence, MRD, ctDNA, MSI-H/dMMR, KRAS, immunotherapy, vaccines, CAR-T/CAR-NK, TCR therapy, and tumor microenvironment.

## 2 Recurrent PDAC as heterogeneous biomarker-defined states

For this review, recurrent PDAC is defined as disease re-emerging after curative-intent treatment. Biomarker interpretation should begin with the recurrence state. Molecular recurrence refers to persistent or newly detectable ctDNA after curative-intent treatment without radiographic disease. Isolated or oligometastatic recurrence describes limited radiographic relapse that may still permit local treatment within a multidisciplinary strategy. Disseminated recurrence includes measurable multi-site disease requiring prompt systemic control, whereas refractory recurrence occurs after multiple treatment lines and is often accompanied by reduced performance status, immune dysfunction, and limited time for complex cellular-product manufacturing. Evidence generated in one state should not be assumed to apply directly to another. This state-based classification is supported by recurrence-risk, post-recurrence survival, and ctDNA MRD literature (3–7).

These distinctions change the plausibility of immune intervention. Vaccines and immune-priming strategies may be most rational in ctDNA-positive MRD or low-volume relapse, where antigen burden is present but immune exclusion and clonal complexity may be less advanced. Checkpoint blockade is clinically justified primarily for MSI-H/dMMR disease. CAR-T, CAR-NK, and TCR-engineered approaches require measurable target expression or HLA-restricted antigen presentation, adequate organ function, sufficient time for manufacturing, and a plan for bridging therapy when disease is rapidly progressive. High-volume or symptomatic recurrence generally requires established cytoreductive treatment, with immune therapy considered within a biomarker-selected trial rather than as an empiric substitute. This risk-matched interpretation is consistent with available data on MSI-H/dMMR checkpoint sensitivity, ctDNA-enriched MRD, vaccine immunogenicity, and adoptive-cell therapy feasibility (5–7, 12–14, 22–29).

ctDNA is one of the most promising tools for detecting recurrence risk after resection, but its role is currently more prognostic than treatment-predictive (5–7). Postoperative ctDNA positivity identifies a population with high residual-disease risk, yet assay sensitivity, tumor-informed versus tumor-naive design, timing, and interlaboratory standardization remain unresolved. A ctDNA-positive result alone should therefore not be interpreted as proof that a vaccine or checkpoint inhibitor will improve outcome. Its immediate value is to enrich prospective MRD trials, define sampling time points, and provide an early molecular endpoint. CA19-9 should likewise be treated as a monitoring marker rather than a validated predictor of benefit from vaccination or adoptive cell therapy.

At radiographic recurrence, tissue and liquid biopsy should be considered complementary. Tissue can assess MSI-H/dMMR status, HLA-dependent antigen presentation, surface-antigen density, and spatial tumor microenvironment features, whereas plasma may capture systemic clonal heterogeneity and serial kinetics. Archival resection specimens may be inadequate when recurrence has emerged after chemotherapy or when antigen loss and microenvironmental remodeling have occurred. Biomarkers must therefore be classified as prognostic, predictive, pharmacodynamic, or resistance-related before they are used for treatment assignment. These biomarker categories are supported by evidence on liquid biopsy, MSI-H/dMMR, HLA-restricted therapy, antigen-directed cell therapy, and spatial TME profiling (5–7, 12–14, 27–33). Here, prognostic biomarkers estimate recurrence risk, monitoring biomarkers track disease burden or treatment response, and treatment-predictive biomarkers identify differential benefit from a defined therapy.

Genomic and liquid-biopsy biomarkers include microsatellite instability-high/mismatch repair deficiency (MSI-H/dMMR), tumor mutational burden (TMB), KRAS alterations, and circulating tumor DNA (ctDNA) kinetics. Tumor-surface and human leukocyte antigen (HLA) profiling can define CLDN18.2, mesothelin, carcinoembryonic antigen (CEA), and intracellular neoantigens for CAR-T/CAR-NK or TCR-engineered strategies. Tumor microenvironment (TME) and spatial profiling can identify myeloid or cancer-associated fibroblast (CAF)-mediated suppression, T-cell dysfunction, and immune exclusion. Evidence maturity is not equivalent across categories: MSI-H/dMMR is clinically actionable; ctDNA-informed MRD strategies, vaccines, CLDN18.2 CAR-T, and KRAS-specific TCR therapy have early clinical or proof-of-concept support; and most immune-state and spatial TME signatures remain exploratory. The framework is intended for treatment selection and trial enrichment, not as evidence that all depicted interventions are standard care. Supporting evidence for the depicted biomarker categories and intervention classes is provided by ctDNA MRD studies, MSI-H/dMMR immunotherapy cohorts, vaccine and cellular-therapy studies, and TME profiling literature (5–43).

## 3 Checkpoint blockade and vaccine strategies: evidence, failures, and biomarker selection

### 3.1 Checkpoint blockade: a rare actionable subgroup within predominantly negative trials

For checkpoint blockade, MSI-H/dMMR status remains the clearest clinically actionable biomarker in recurrent PDAC. A European cohort of MSI-H/dMMR advanced PDAC reported meaningful responses to immune checkpoint inhibitors, and plasma-based detection may be useful when tissue is limited (12, 13). However, MSI-H/dMMR represents only a small minority of PDAC. Programmed death-ligand 1 expression alone has not reliably selected responders, and tumor mutational burden without a corresponding assessment of neoantigen clonality, antigen presentation, and immune context is insufficient.

Negative trials define the current boundary of the field. In 27 patients with locally advanced or metastatic pancreatic adenocarcinoma, ipilimumab monotherapy produced no conventional objective responses (8). In a randomized phase 2 study, durvalumab monotherapy produced no responses and durvalumab plus tremelimumab achieved an objective response rate of 3.1%, below the prespecified threshold for further study (10). In PA.7, adding durvalumab and tremelimumab to gemcitabine plus nab-paclitaxel did not improve overall survival, progression-free survival, or response rate in an unselected metastatic population (11). PRINCE suggested that treatment-specific immune states may matter, but its primary endpoint used a historical benchmark, the study was not powered for between-arm comparisons, and the biomarker analyses were exploratory (9).

Checkpoint blockade has failed in most PDAC patients because several steps of the cancer-immunity cycle are impaired simultaneously. Many tumors have a low burden of clonally expressed neoantigens; antigen processing and HLA class I presentation may be reduced; effector T cells are sparse or spatially excluded; and CAFs, tumor-associated macrophages, myeloid-derived suppressor cells, regulatory T cells, TGF-*β* signaling, hypoxia, and nutrient competition suppress immune function (17–19, 30–39). Rapid progression and high tumor burden can outpace immune priming, while prior chemotherapy may reduce lymphocyte fitness. These interacting mechanisms explain why a single marker such as PD-L1 or a single checkpoint inhibitor is unlikely to be sufficient. Biomarkers should therefore identify the dominant resistance mechanism and be linked to a treatment designed to address it. Thus, clinical failure reflects a combined defect in antigen generation or presentation, effector-cell recruitment, effector-cell function, stromal access, and treatment timing rather than failure of a single checkpoint pathway alone (8–11, 15–21, 30–43).

### 3.2 Vaccine-based immunotherapy: strongest rationale in molecular or low-volume recurrence

Personalized RNA neoantigen vaccination has shown that resected PDAC can generate durable vaccine-induced T-cell responses in a subset of patients (22, 23). Its principal strength is patient-specific targeting of expressed neoantigens; its limitations include the need for adequate tissue, sequencing and epitope prediction, HLA-informed manufacturing, production time, and uncertainty regarding whether immune responses translate into improved recurrence-free or overall survival. The available evidence is derived mainly from resected patients rather than established recurrent PDAC. Accordingly, personalized vaccination is most plausibly tested in ctDNA-positive MRD or low-volume relapse, not in rapidly progressive high-burden disease.

KRAS-directed vaccination offers a more standardized strategy because recurrent hotspot mutations are common in PDAC. AMPLIFY-201 demonstrated immunogenicity and encouraging molecular-disease observations in biomarker-selected patients, and a phase 1 study combining mutant KRAS vaccination with checkpoint blockade supports further investigation (24–26). However, the trials are early phase, include mixed tumor types or post-resection populations, and do not establish benefit in radiographically recurrent PDAC. KRAS genotype, HLA restriction, baseline ctDNA, vaccine-induced T-cell expansion, and ctDNA clearance should therefore be prespecified as selection and pharmacodynamic variables. Among vaccine approaches, the near-term opportunity is trial enrollment in molecularly recurrent or low-volume disease, where manufacturing time and immune priming are clinically feasible.

## 4 Biomarkers guiding adoptive cell therapy: comparative evidence and constraints

Adoptive cell therapy requires a different biomarker logic from checkpoint blockade. The primary questions are whether tumor cells express a safe and sufficiently homogeneous target, whether antigen processing and HLA presentation are intact for TCR-engineered therapy, whether infused cells can traffic into tumor deposits, and whether the patient can tolerate lymphodepletion and treatment-related toxicity (27–29, 44–47). Target positivity by archival tissue alone is insufficient; antigen density, spatial heterogeneity, metastatic-site concordance, and the possibility of antigen-negative escape should be considered whenever feasible.

CLDN18.2-directed CAR-T therapy currently provides one of the strongest early clinical signals among gastrointestinal solid tumors, but pancreatic-specific evidence remains limited. A phase 1 study in a mixed gastrointestinal cancer cohort demonstrated activity, whereas a recurrent PDAC case report described complete remission followed by antigen-negative relapse (27, 28). The response supports biological activity, but the single-case design and subsequent target loss prevent broad generalization. CLDN18.2 testing should therefore be treated as a trial-enrollment biomarker, with predefined expression thresholds and serial assessment when progression occurs. Evidence for mesothelin, CEA, HER2, MUC1, and B7-H3 in recurrent PDAC is less mature and remains predominantly early phase or preclinical.

TCR-engineered therapy expands the target space to intracellular mutation-derived epitopes. A single-patient report of objective regression after KRAS G12D-reactive TCR gene therapy provides proof-of-concept that a driver neoantigen can be therapeutically targeted in metastatic PDAC (29). The approach is nevertheless restricted by exact HLA compatibility, epitope presentation, tumor clonality, manufacturing complexity, immune fitness, and off-target safety. A single exceptional response should not be interpreted as an established treatment effect. Candidate selection requires paired tumor genotyping, high-resolution HLA typing, confirmation of antigen presentation when possible, and a realistic assessment of the time needed to produce the cell product.

CAR-NK therapy offers potential advantages, including allogeneic or off-the-shelf production, innate cytotoxicity, and possibly lower rates of some T-cell-associated toxicities (46, 47). Its disadvantages include uncertain persistence, predominantly preclinical evidence and very limited PDAC-specific clinical data, and susceptibility to the same stromal, metabolic, and trafficking barriers that constrain CAR-T cells. At present, CAR-NK biomarkers are principally research tools: target-antigen density, NK-cell ligands, inhibitory receptor profiles, cytokine support, and *in vivo* persistence should be embedded in early-phase trials.

Across platforms, no adoptive cell therapy is standard care for recurrent PDAC. CAR-T is most developed for accessible surface antigens but is vulnerable to heterogeneous expression and target loss. TCR-engineered therapy can reach intracellular driver neoantigens but applies only to specific HLA-epitope pairs. CAR-NK may improve scalability but has the least mature disease-specific evidence. The most appropriate candidates are fit patients with biomarker-confirmed targets, limited or controllable disease burden, access to specialized centers, and a feasible bridging strategy. These therapies should be presented as clinical-trial options rather than routine alternatives to established systemic treatment.

## 5 Tumor microenvironment biomarkers: biological value and clinical limitations

The PDAC tumor microenvironment (TME) is a central barrier to immunotherapy. Single-cell and spatial studies show that malignant-cell state, treatment exposure, fibroblast phenotype, myeloid composition, and lymphocyte localization jointly shape immune response (30–33). These technologies add information beyond bulk lymphocyte counts: the distance of CD8 + T cells from malignant glands, the density and phenotype of cancer-associated fibroblasts (CAFs), tumor-associated macrophages (TAMs), myeloid-derived suppressor cells (MDSCs), and regulatory T cells (Tregs), and local hypoxic or cytokine gradients may determine whether immune cells can reach and function within recurrent lesions.

These biomarkers are biologically compelling but clinically immature. CAF and macrophage states are heterogeneous, can have context-dependent functions, and may vary between primary tumors and recurrent metastatic sites (34–39). Spatial assays are sensitive to sampling location, tissue quality, antibody panels, segmentation methods, and analytic thresholds. Most reported signatures are retrospective or correlative, and no CAF-, TAM-, or spatial-exclusion signature is currently a validated companion diagnostic for recurrent PDAC. Indiscriminate stromal depletion may also remove tumor-restraining elements, underscoring the need for mechanism-specific rather than generic TME modulation.

For trial design, TME profiling should answer a defined therapeutic question. Preserved antigen expression with marked CAF/myeloid exclusion may support a cell-therapy trial combined with a mechanism-matched remodeling agent. An inflamed MSI-H/dMMR tumor may support checkpoint blockade without additional stromal intervention. ctDNA-positive MRD with no measurable lesion is poorly suited to tissue-intensive spatial profiling but may be appropriate for vaccine-based immune priming. Among current approaches, multiplex immunohistochemistry and focused transcriptomic signatures are more feasible than comprehensive spatial multi-omics, but all require prospective validation against treatment-specific outcomes.

TME biomarkers should therefore be used primarily for clinical-trial enrichment, pharmacodynamic assessment, and resistance analysis. Their value lies in identifying why a patient is unlikely to respond to immune activation alone, not in creating an unvalidated universal score. Cross-platform harmonization, metastatic-site sampling, and serial assessment before and after therapy are necessary before these measures can guide routine care.

## 6 A practical biomarker-guided workflow for recurrent PDAC trials

Distinguish ctDNA-defined MRD, isolated or oligometastatic relapse, disseminated measurable recurrence, and refractory progression. Record recurrence-free interval, disease tempo, symptoms, prior treatment, performance status, and whether immediate cytoreduction is required. Confirm MSI-H/dMMR status, perform broad tumor or plasma genomic profiling, document KRAS genotype, and assess a relevant surface antigen or HLA-restricted epitope only when a corresponding trial is available. Tissue at recurrence is preferred when safe and feasible; archival tissue and ctDNA should be interpreted as complementary rather than interchangeable. MSI-H/dMMR disease supports checkpoint blockade in clinical practice. ctDNA-defined MRD, KRAS or personalized vaccines, CAR-T/CAR-NK therapy, TCR-engineered therapy, and TME-remodeling combinations should be pursued within biomarker-selected trials. PD-L1, TMB, immune signatures, or spatial TME features should not be used as stand-alone reasons for off-label checkpoint therapy in microsatellite-stable PDAC. Evaluate organ function, inflammatory and nutritional status, infection risk, tissue availability, HLA eligibility, manufacturing time, referral distance, cost, regulatory access, and the need for bridging therapy. A biologically suitable target is not clinically actionable if the patient is likely to progress before product delivery or cannot safely receive lymphodepletion. Combine imaging and carbohydrate antigen 19–9 (CA19-9) with ctDNA kinetics when informative. For vaccines and TCR-based approaches, track antigen-specific immune responses; for CAR-based therapy, assess cell expansion and persistence; and at progression, evaluate antigen loss, HLA or antigen-presentation changes, and TME remodeling when re-biopsy is feasible. This workflow is supported by the cited evidence on recurrence kinetics, actionable genomics, vaccine and cell-therapy platforms, and TME-based resistance mechanisms (5–43).

**Figure 1:**
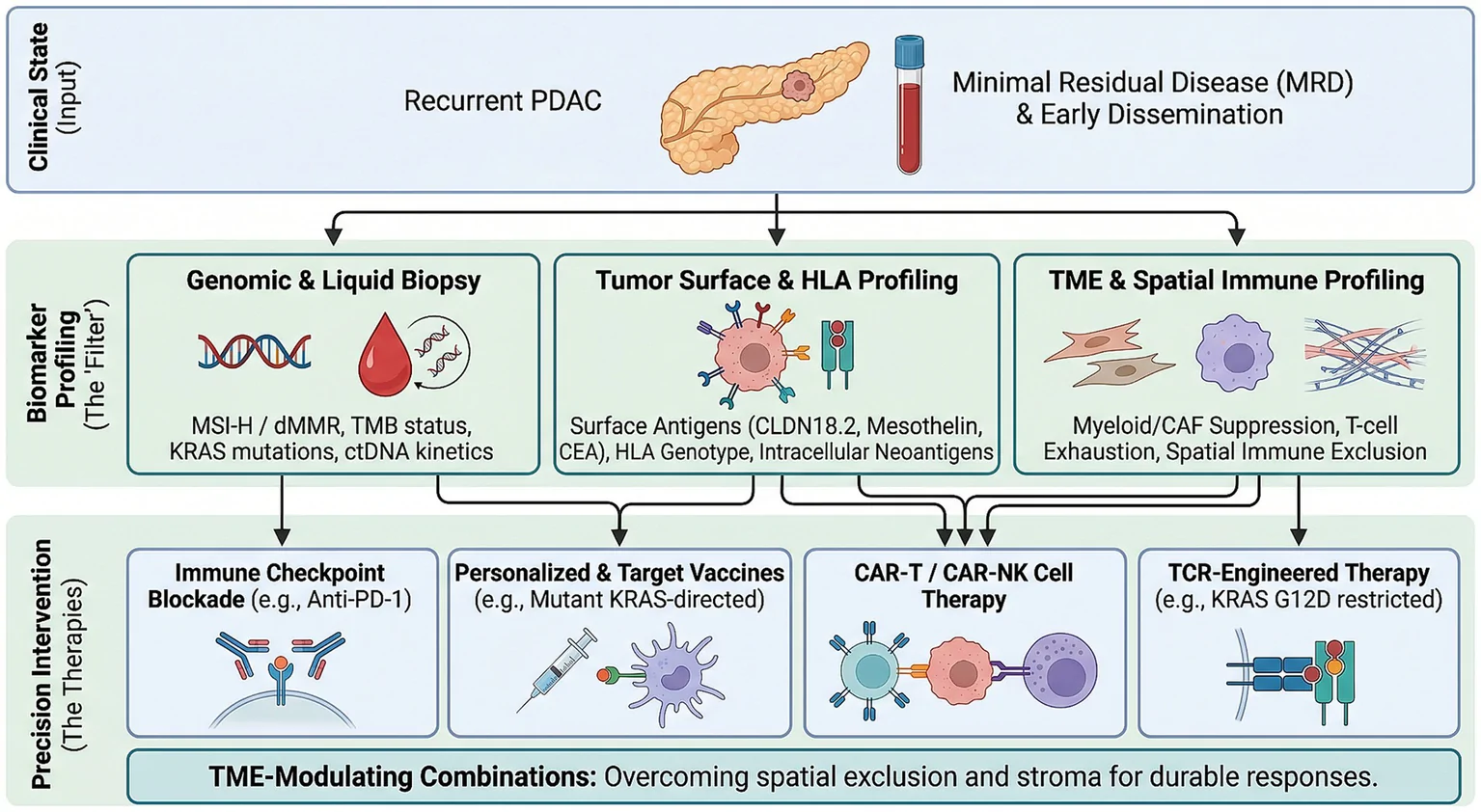
Biomarker-driven decision framework for immunotherapy and adoptive cell therapy in recurrent pancreatic ductal adenocarcinoma. The clinical sequence is: (1) Classify the recurrence state as molecular residual disease (MRD), limited radiographic relapse, disseminated recurrence, or refractory disease; (2) obtain a minimum biomarker panel using tissue and/or liquid biopsy; (3) match the patient to an evidence-appropriate intervention; (4) assess feasibility, including performance status, organ function, tissue availability, manufacturing time, access, and need for bridging therapy; and (5) monitor response and immune escape longitudinally.

This workflow reduces repetition by assigning each biomarker a specific function: recurrence detection, treatment selection, feasibility assessment, pharmacodynamic monitoring, or resistance analysis. Figure 1 summarizes the decision sequence, and Table 1 distinguishes current clinical applicability from early-phase and exploratory evidence.

**Table 1:** Evidence maturity and biomarker-guided use in recurrent PDAC.

Biomarker domain	Representative biomarkers or measurements	Evidence status and current clinical applicability	Biomarker-guided use and principal validation need
MRD and recurrence kinetics	ctDNA, CA19-9, imaging pattern, time to recurrence	Prospective and retrospective prognostic evidence; ctDNA is not yet a validated predictive trigger for immune therapy.	Enrich low-burden or MRD trials and guide monitoring; harmonize assays and sampling and prove that ctDNA-triggered intervention improves outcomes.
Actionable immunotherapy genomics	MSI-H/dMMR, TMB, KRAS genotype, KRAS mutant dosage, neoantigen quality	MSI-H/dMMR is clinically actionable for checkpoint blockade; TMB, KRAS dosage, and neoantigen metrics remain investigational in PDAC.	Use MSI-H/dMMR in routine selection and other features for trial matching; validate tissue-plasma concordance and treatment-specific predictive value.
Target-antigen expression	CLDN18.2, mesothelin, CEA, HER2, MUC1, B7-H3	Early-phase clinical signal for CLDN18.2 CAR-T across gastrointestinal cancers; pancreatic-specific evidence is limited and other targets are earlier stage.	Select CAR-T or CAR-NK trials; define antigen-density thresholds, spatial heterogeneity, off-tumor risk, and post-treatment antigen loss.
Antigen presentation and TCR eligibility	HLA type, B2M, TAP1/TAP2, mutation-derived epitopes	Case-based or small early-phase proof-of-concept; no established standard for recurrent PDAC.	Match HLA-restricted TCR trials; confirm epitope presentation, clonality, manufacturing feasibility, and safety.
Effector immune state	CD8 + T cells, IFN-γ signatures, GZMB, PRF1, NKG7, T-cell clonality	Exploratory correlative evidence from translational cohorts; no validated companion diagnostic.	Stratify immune-priming and monitoring studies; prospectively validate assay cutoffs and dynamic associations with response.
Suppressive and spatial TME	CAF, TAM, MDSC, Treg, TGF-β, CXCL12, hypoxia, immune exclusion	Strong preclinical and translational rationale, but spatial signatures are not clinically validated.	Enrich mechanism-matched combination trials; harmonize multiplex or spatial assays and demonstrate treatment-specific predictive value.
Host and toxicity risk	Performance status, inflammatory markers, cytokines, organ function, prior therapy	Performance status and organ function are routine eligibility factors; cytokine and toxicity models remain exploratory.	Assess fitness, bridging therapy, and monitoring intensity; validate prospective toxicity prediction and standardized adverse-event annotation.

Evidence categories are based on the clinical and translational studies summarized in the main text (5–43). B2M, beta-2-microglobulin; CAF, cancer-associated fibroblast; CAR-T, chimeric antigen receptor T-cell; CAR-NK, chimeric antigen receptor natural killer-cell; CEA, carcinoembryonic antigen; ctDNA, circulating tumor DNA; HLA, human leukocyte antigen; IFN-γ, interferon gamma; MDSC, myeloid-derived suppressor cell; MRD, molecular residual disease; MSI-H/dMMR, microsatellite instability-high/mismatch repair-deficient; TAM, tumor-associated macrophage; TCR, T-cell receptor; TGF-β, transforming growth factor beta; TMB, tumor mutational burden; Treg, regulatory T cell. Evidence status: clinically actionable = used for current PDAC treatment selection; early clinical = phase I/II, mixed-cohort, or case-level human evidence not establishing standard care; exploratory = retrospective, translational, or preclinical evidence requiring prospective validation.

## 7 Discussion

The central conclusion of this Mini Review is that biomarker-driven immunotherapy in recurrent PDAC should be framed by evidence boundaries rather than technological availability. Negative checkpoint trials demonstrate that unselected PDAC is not rescued by simply adding immune checkpoint blockade to chemotherapy (8–11). The clinically actionable exception is MSI-H/dMMR disease. Beyond that subgroup, the strongest rationale is not for empiric immunotherapy but for mechanism-matched trials in which recurrence state, target biology, immune context, and treatment feasibility are specified prospectively.

The relative readiness of emerging approaches differs substantially. ctDNA has increasing prognostic value for postoperative residual disease but is not yet a validated predictive trigger for a particular immune treatment. Personalized and KRAS-directed vaccines have generated convincing immunogenicity, yet survival benefit in recurrent PDAC remains unproven and the clearest testing opportunity is low-burden or molecular relapse. CLDN18.2 CAR-T and KRAS-specific TCR therapy provide early clinical proof-of-concept, but pancreatic-specific sample sizes are small and selection is constrained by antigen heterogeneity, HLA restriction, manufacturing, toxicity, and access. CAR-NK platforms and spatial TME signatures remain earlier in development (22–26).

Several limitations affect the underlying evidence and this review. Most studies are early phase, single-arm, retrospective, case-based, or exploratory; many enroll resected or first-line metastatic populations rather than patients with clearly defined recurrent PDAC. Cross-study comparisons are limited by different assays, sampling times, recurrence definitions, prior treatments, and endpoints. Recurrent lesions are not always biopsied, archival tissue may not represent current antigen or TME status, and ctDNA sensitivity varies with disease volume and shedding. This Mini Review is a focused narrative synthesis and does not provide a quantitative meta-analysis; rapid publication in this field also means that evidence maturity may change.

Implementation poses additional barriers. Comprehensive genomic, HLA, antigen, and spatial profiling may not be available at all centers, turnaround time can exceed the therapeutic window of rapidly progressive disease, and cellular therapy requires referral networks, specialized manufacturing, bridging treatment, toxicity management, and financial support. Re-biopsy may be unsafe, low-shedding disease may yield false-negative plasma results, and assay platforms lack harmonized thresholds. Multidisciplinary molecular tumor boards are needed to distinguish standard-of-care findings from trial-only hypotheses and to prevent overinterpretation of exploratory biomarkers (27–29, 44–47).

Future recurrent-PDAC trials should predefine the recurrence state, use a minimum harmonized biomarker set, and separate prognostic from predictive endpoints. Randomized or adaptive designs should include standard-therapy comparators when feasible, prospectively collect tissue and plasma, measure treatment-specific pharmacodynamics, and analyze mechanisms of nonresponse and escape. The immediate goal is not to replace chemotherapy, surgery, or radiotherapy, but to identify the limited clinical states in which an immune intervention can be added rationally and tested rigorously.

## 8 Conclusion

Recurrent PDAC requires a restrained, evidence-tiered precision immuno-oncology strategy. Checkpoint blockade is clinically justified for MSI-H/dMMR tumors, whereas vaccines, CAR-T/CAR-NK cells, TCR-engineered therapy, and TME-modulating combinations remain investigational. Their development should prioritize molecular or low-volume recurrence when immune priming and manufacturing are feasible, require biomarker-confirmed eligibility, and incorporate longitudinal assessment of ctDNA, target expression, immune activity, and resistance. A biomarker is useful only when it changes a defined clinical or trial decision. Applying this principle can convert a descriptive biomarker catalog into a testable framework for recurrent PDAC.
